# Double Crystallization and Phase Separation in Polyethylene—Syndiotactic Polypropylene Di-Block Copolymers

**DOI:** 10.3390/polym13162589

**Published:** 2021-08-04

**Authors:** Claudio De Rosa, Rocco Di Girolamo, Alessandra Cicolella, Giovanni Talarico, Miriam Scoti

**Affiliations:** Dipartimento di Scienze Chimiche, Università di Napoli Federico II, Complesso Monte S. Angelo, Via Cintia, I-80126 Naples, Italy; rocco.digirolamo@unina.it (R.D.G.); alessandra.cicolella@unina.it (A.C.); talarico@unina.it (G.T.); miriam.scoti@unina.it (M.S.)

**Keywords:** semicrystalline block copolymers, phase separation and crystallization, epitaxial crystallization, nanostructures

## Abstract

Crystallization and phase separation in the melt in semicrystalline block copolymers (BCPs) compete in defining the final solid state structure and morphology. In crystalline–crystalline di-block copolymers the sequence of crystallization of the two blocks plays a definitive role. In this work we show that the use of epitaxial crystallization on selected crystalline substrates allows achieving of a control over the crystallization of the blocks by inducing crystal orientations of the different crystalline phases and a final control over the global morphology. A sample of polyethylene-*block*-syndiotactic polypropylene (PE-*b*-sPP) block copolymers has been synthesized with a stereoselective living organometallic catalyst and epitaxially crystallized onto crystals of two different crystalline substrates, p-terphenyl (3Ph) and benzoic acid (BA). The epitaxial crystallization on both substrates produces formation of highly ordered morphologies with crystalline lamellae of sPP and PE highly oriented along one direction. However, the epitaxial crystallization onto 3Ph should generate a single orientation of sPP crystalline lamellae highly aligned along one direction and a double orientation of PE lamellae, whereas BA crystals should induce high orientation of only PE crystalline lamellae. Thanks to the use of the two selective substrates, the final morphology reveals the sequence of crystallization events during cooling from the melt and what is the dominant event that drives the final morphology. The observed single orientation of both crystalline PE and sPP phases on both substrates, indeed, indicates that sPP crystallizes first onto 3Ph defining the overall morphology and PE crystallizes after sPP in the confined interlamellar sPP regions. Instead, PE crystallizes first onto BA defining the overall morphology and sPP crystallizes after PE in the confined interlamellar PE regions. This allows for discriminating between the different crystalline phases and defining the final morphology, which depends on which polymer block crystallizes first on the substrate. This work also shows that the use of epitaxial crystallization and the choice of suitable substrate offer a means to produce oriented nanostructures and morphologies of block copolymers depending on the composition and the substrates.

## 1. Introduction

In semicrystalline block copolymers (BCPs) microphase separation arises from incompatibility of the blocks as in amorphous BCPs, or by crystallization of one or more blocks [1]. Microphase separation in the melt of dissimilar blocks and crystallization may compete and generate a wide range of morphologies [1,2,3,4,5,6,7]. The final morphology is path dependent and is the result of this competition and of the interplay between phase separation of the incompatible blocks and the crystallization of blocks [1,2,3,4,5,6,7]. Different morphologies are possible depending on the composition of the BCP, the crystallization and glass transition temperatures of blocks and the order–disorder transition temperature. Various structures are obtained depending on which process between crystallization and phase separation occurs first [8]. When crystallization occurs first, from a homogeneous melt, it drives the microphase separation and the final structure is defined by the crystal morphology. If microphase separation occurs first, crystallization occurs from a microphase separated heterogeneous melt, resulting in a crystallization confined within preformed microdomains, or breaking out of the microphase separated structure formed in the melt [7,8,9,10,11,12,13,14,15,16,17,18,19]. In crystalline–crystalline block copolymers the crystallization of the first block may define the final morphology or be modified by the subsequent crystallization of the other block [20,21,22,23,24].

BCPs containing blocks based on crystallizable stereoregular polyolefins have been synthesized only recently thanks to the development of metal-based insertion polymerization methods able to ensure a high stereochemical control in living olefin polymerization [25], and studies on the crystallization and phase separation of BCPs containing linear polyethylene and isotactic and syndiotactic polypropylene have been published [18,19,23,24,26,27].

Crystallizable block copolymers have been mainly studied in the past for their possible application as thermoplastic elastomers due to their improved mechanical properties and better thermal stability. Moreover, the presence of a crystallizable component can be exploited for controlling the final morphology through the control of crystallization and orientation of the crystals [7]. In particular, a method for controlling the crystallization and crystal orientation of semicrystalline polymers in thin films is the epitaxial crystallization on suitable crystalline substrates [28]. This method allows the inducing of preferred orientation of crystals of polymers on the substrate and/or crystallization of unstable crystal modifications [28]. Driving crystallization of specific polymorphic forms of polymers is of interest to tailor materials’ properties [29]. Recently this method has been applied to crystalline BCPs [7], resulting in the formation of highly ordered nanostructures with highly aligned microdomains as a consequence of the orientation of the crystalline phase [7,15,16,17,18,19,23,24].

In this paper we report a study of the structure and morphology of a crystalline–crystalline BCP composed of blocks of crystallizable polyethylene (PE) and syndiotactic polypropylene (sPP) (PE-b-sPP). The two crystallizable PE and sPP components have been epitaxially crystallized on two different crystalline substrates, that is, crystals of *p*-terphenyl (3Ph) and benzoic acid (BA). The two different substrates induce selective and different orientations of the two PE and sPP crystalline phases with a final morphology composed of highly aligned lamellar domains with long crystalline sPP and PE lamellae aligned along one direction. Thanks to the use of the two selective substrates, the final morphology reveals the sequence of crystallization events during cooling from the melt and what is the dominant event that drives the final morphology. We also show that use of epitaxial crystallization and the choice of suitable substrate offer a means to produce different oriented nanostructures and morphologies of BCPs depending on the BCP composition and the substrates.

## 2. Materials and Methods

The sample of PE-b-sPP was prepared with a living organometallic catalyst, bis[N-(3-tert-butylsalicylidene)-2,3,4,5,6-pentafluoroanilinato]-titanium(IV) dichloride (from MCAT, Donaueschingen, Germany), activated with methylalumoxane (MAO) (from Lanxess, Cologne, Germany) [30,31]. The molecular mass and the polydispersity of the sample was determined by gel permeation chromatography (GPC), using a Polymer Laboratories GPC220 apparatus equipped with a Viscotek 220R viscometer (Agilent Company, Santa Clara CA, USA), on polymer solutions in 1,2,4-trichlorobenzene at 135 °C. The molecular structure was analyzed by ^13^C NMR spectroscopy using a Varian VXR 200 spectometer (Varian Company, Palo Alto, CA, USA).

The sample PE-*b*-sPP has a total molecular mass *M*_n_ = 22,000 g/mol with *M*_w_/*M*_n_ = 1.2 and a sPP block longer than the PE block (*M*_n(sPP)_ = 18,900 and *M*_n(PE)_ = 3100) with 20 mol% of ethylene, evaluated from ^13^C NMR spectrum (corresponding to 14 wt% of PE). The molecular mass of the blocks was estimated from total *M*_n_ and wt% of PE or sPP, such that *M*_n(PE)_ = *M*_n_ × 0.14 ≈ 3,100 g/mol and *M*_n(sPP)_ = *M*_n_ − *M*_n(PE)_ ≈ 18,900 g/mol. The volume fraction of the PE block is *f*_PE_ = 13% and was calculated from the molecular masses *M*_n(PE)_ and *M*_n(sPP)_ and the densities of PE (0.997 g/cm^3^) and sPP (0.9 g/cm^3^) [32] such that *f*_PE_ = (*M*_n(PE)_ / 0.997) / (*M*_n(sPP)_ / 0.9 + *M*_n(PE)_ / 0.997). The ^13^C NMR spectrum and the GPC trace of the sample PE-*b*-sPP are reported in the supporting information.

It is worth noting that the sample PE-*b*-sPP analyzed in this paper is different in terms of molecular mass and relative lengths of PE and sPP blocks from the samples reported in our previous paper [23]. The sample PE-*b*-sPP has, indeed, a PE block much shorter than the sPP block with 13% volume fraction of PE, whereas in [23] a nearly symmetric sample with *f*_PE_ = 47% and a sample with higher molecular mass and *f*_PE_ = 25% were analyzed.

Calorimetric measurements (DSC-822, Mettler Toledo, Columbus, OH, USA) were performed under flowing N_2_ at heating and cooling rates of 10 °C/min. X-ray powder diffraction profiles were obtained with Ni-filtered CuKα radiation with X-Pert diffractometer (Panalytical, Malvern, UK). Diffraction profiles were also recorded in situ at different temperatures during heating and cooling from the melt at about 10 °C/min using an attached TTK Anton-Paar non-ambient stage. The sample was heated from 25 °C up to the melt at 150 °C at nearly 10 °C/min and the diffraction profiles were recorded every 5 degrees starting from 105 °C up to 150 °C. Then, the sample was cooled from the melt at 150 °C down to 25 °C still at 10 °C/min and the diffraction profiles were recorded every 5 degrees during cooling. The temperature was kept constant during recording of each diffraction profile during both heating and cooling.

**Figure 1:**
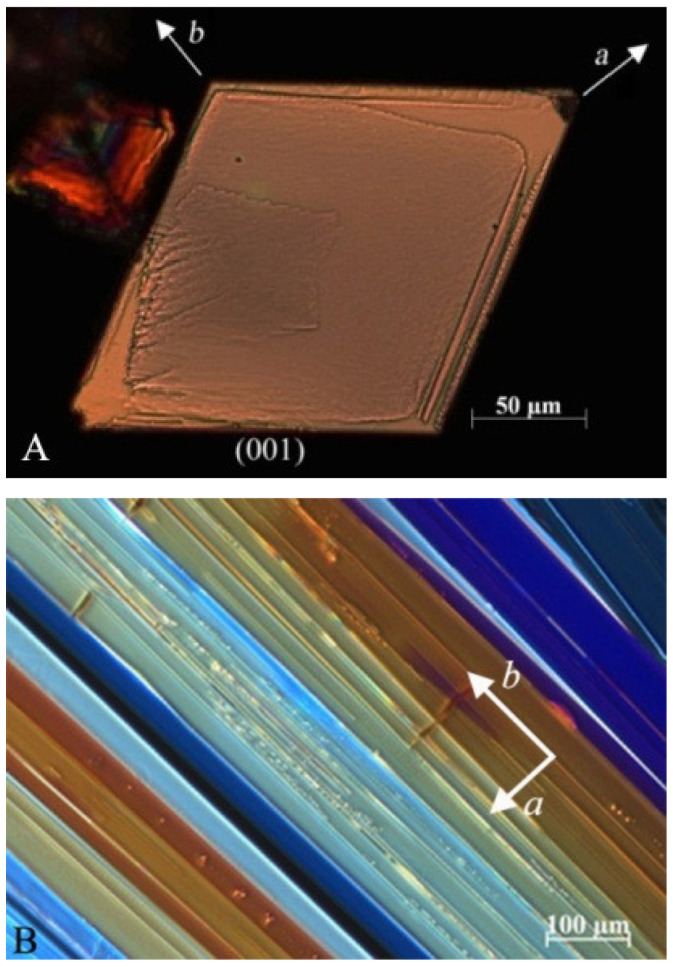
Polarized optical microscope images of flat crystal of 3Ph with exposed (001) face (**A**) and of directionally crystallized flat BA crystals (**B**). BA crystals are elongated and aligned with the b axis parallel to growth front direction. BA single crystals with various thicknesses lead to different colors under polarized light [37].

Epitaxial crystallizations of the block copolymer on the crystals of *p*-terphenyl (3Ph) or benzoic acid (BA) were performed following the procedure used for the PE [33,34,35] and sPP [36] homopolymers. Thin films (thickness lower than 50 nm) of the BCP were cast at room temperature on microscope glass slides from a *p*-xylene solution (0.2 wt%–0.5 wt%). Slightly different procedures were used for producing crystals of 3Ph and BA substrates. Single crystals of 3Ph were produced independently by slow cooling of a boiling acetone solution; a drop of the suspension was deposited onto the polymer film at room temperature. After evaporation of the solvent, large (≈ 10–100 μm), flat crystals of 3Ph delimited by large top and bottom (001) surfaces remain on the copolymer film (Figure 1A). This composite material was heated to ≈ 180 °C to melt the sPP and PE for a short time to limit sublimation of the 3Ph substrate, and then recrystallized by cooling at a controlled rate (10–15 °C/min) to room temperature. During cooling sPP and PE crystallize epitaxially at the interface with the 3Ph crystals. The 3Ph crystals were subsequently dissolved with hot acetone. In the case of BA, powder of BA was spread on the BCP films; then, the polymer film was melted along with BA (melting temperature of BA equal to 123 °C) at ≈180 °C to melt both the BCP and BA and then the mixtures were crystallized by moving the slide slowly down the temperature gradient of a hot bar (cooling rate 10–15 °C/min). On cooling, the BA substrate crystals grow first through directional crystallization forming large, flat, and elongated crystals aligned with the *b* axis parallel to the growth front direction (Figure 1B) [35]. Then, the polymer crystallizes at lower temperatures epitaxially onto the (001) exposed face of BA crystals. These crystals of BA were subsequently dissolved with hot ethanol and the polymer film left on the glass.

The so obtained thin films crystallized onto 3Ph and BA were carbon-coated under vacuum in an EMITECH K950X evaporator (Quorum Technologies, Lewes, UK). To improve contrast, the thin films were decorated with gold nanoparticles by vacuum evaporation and condensation. After evaporation, gold condensates and deposits mainly at amorphous–crystalline interface of the semicrystalline lamellae, allowing better visualization of crystalline phases. The films were then floated off on water with the help of a poly(acrylic acid) backing and mounted on copper grids. Transmission electron microscope (TEM) images in bright-field mode were taken in a FEI TECNAI G^2^ 200kV S-TWIN microscope (electron source with LaB_6_ emitter) (FEI Company, Dawson Creek Drive, Hillsboro, OR, USA). Bright-field (BF) TEM images were acquired at 120 or 200 kV. 

## 3. Results and Discussion

**Figure 2:**
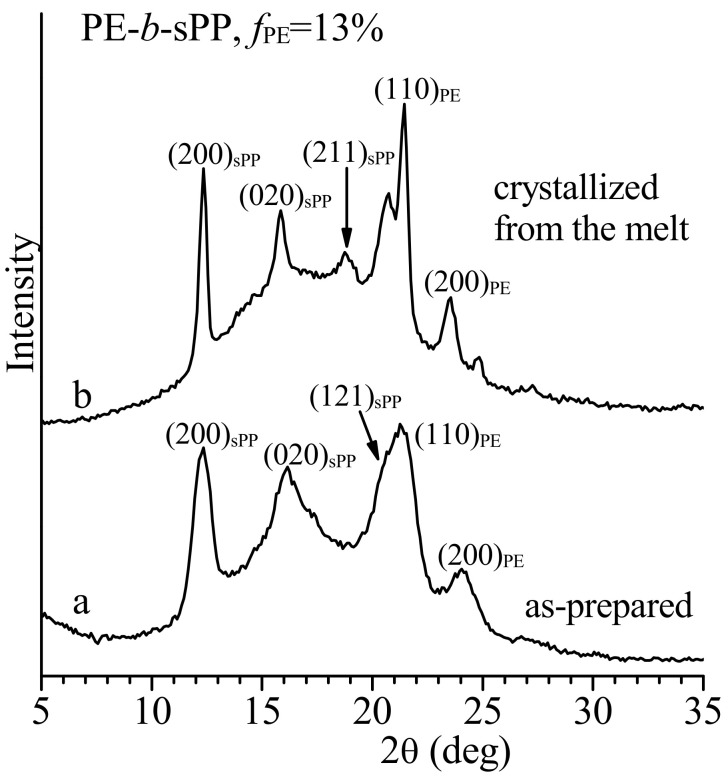
X-ray powder diffraction profiles of as-prepared specimen (a) and of sample crystallized from the melt by cooling the melt at 10 °C/min (b) of the BCP sample PE-*b*-sPP with *f*_PE_ = 13%. The (200)_sPP_, (020)_sPP_, (211)_sPP_ and (121)_sPP_ reflections of form I of sPP at 2θ = 12.2°, 16°, 18.8° and 20.7° and the (110)_PE_ and (200)_PE_ reflections at 2θ = 21.4° and 23.9° of the orthorhombic form of PE are indicated.

The X-ray powder diffraction profile of the as-polymerized sample PE-*b*-sPP is reported in Figure 2. The diffraction profile shows the 200, 020 and 121 reflections of form I of sPP at 2θ = 12.2°, 16° and 20.7° [38,39] and the 110 and 200 reflections at 2θ = 21.4° and 23.9° of the orthorhombic form of PE [40] (profile a of Figure 2). This indicates that PE and sPP blocks crystallize in their most stable polymorphic forms with a total degree of crystallinity of nearly 40%.

**Figure 3:**
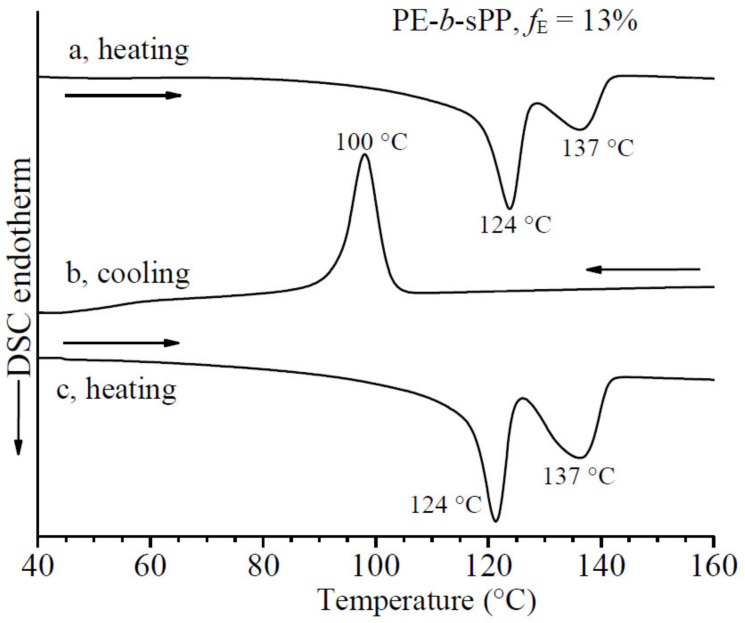
DSC thermograms of the sample PE-*b*-sPP with *f*_PE_ = 13% recorded at scanning rate of 10 °C/min during heating of the as-prepared sample (a), cooling from the melt to room temperature (b) and successive heating of the melt-crystallized sample (c).

The DSC thermograms of the sample PE-*b*-sPP recorded during first heating, successive cooling from the melt and second heating of the melt-crystallized samples, all recorded at 10 °C/min, are reported in Figure 3. The DSC heating curve of the as-prepared sample shows two melting peaks at 124 and 137 °C, which can probably be attributed to the melting of PE at low temperature and of sPP at high temperature. This agrees with the melting temperature of 144 °C (data not shown) of the sPP homopolymer synthesized with the same catalyst and in the same reaction conditions, consistent with a concentration of the syndiotactic pentad *rrrr* of 91%. Since a similar stereoregularity is expected for the PE-*b*-sPP copolymer, the slightly lower melting temperature (137 °C) is probably due to confinement phenomena due to phase separation, or confined crystallization inside crystalline lamellae of the other component [23,31].

It is worth noting that in our previous paper [23] different samples of PE-*b*-sPP BCPs with different relative lengths of PE and sPP blocks have shown only one broad melting peak due to the overlapping of PE and sPP melting. The shorter PE block of the sample here analyzed has been suitably designed to separate the melting endotherms of PE and sPP crystals, as actually occurs in the DSC heating curve of Figure 3a. However, also for this sample the DSC cooling curve from the melt shows only one crystallization peak (curve b of Figure 3), indicating overlapping of crystallization of PE and sPP blocks.

The X-ray diffraction profile of the sample crystallized from the melt in DSC at a cooling rate of 10 °C/min is shown in Figure 2 (profile b). The diffraction profile of Figure 2b shows the 200, 020 and 121 reflections of form I of sPP at 2θ = 12.2°, 16° and 20.7° and the 110 and 200 reflections at 2θ = 21.4° and 23.9° of the orthorhombic form of PE, which are sharper than those in the diffraction profile of the as-prepared sample of Figure 2a. The degree of crystallinity of the melt-crystallized sample is only slightly lower than that of the as-prepared sample (about 40%). Moreover, the diffraction profile of the melt-crystallized sample of Figure 2b shows, in addition, the presence of the 211 reflection at 2θ = 18.8°, typical of the ordered form I of sPP [38,39,41]. This indicates that the crystallization from the melt induces the crystallization of a more ordered modification of form I of sPP, characterized by a more ordered alternation of right-handed and left-handed 2/1 helical chains of sPP along the *a* and *b* axes of the orthorhombic unit cell of form I [39,41]. The absence of the 211 reflection in the diffraction profile of the as-prepared sample of Figure 2a indicates that this sample is instead crystallized in a disordered modification of form I characterized by disorder in the perfect alternation of enantiomophous helices along both axes of the unit cell [38,39,41].

The DSC melting curve of the melt-crystallized sample of Figure 3c still shows two separate melting endotherms at 124 and 137 °C of PE and sPP, respectively.

**Figure 4:**
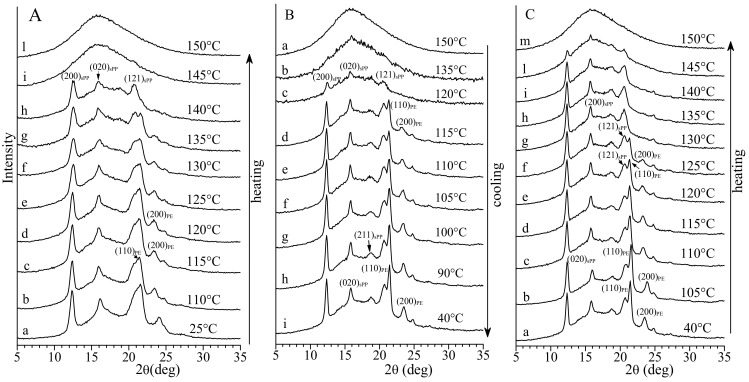
X-ray powder diffraction profiles of the sample PE-*b*-sPP with *f*_PE_ = 13% recorded at different temperatures during first heating of the as-prepared sample up to the melt (**A**), during cooling from the melt to room temperature (**B**) and during successive heating of the melt-crystallized sample up to the melt (**C**). The (200)_sPP_, (020)_sPP_, (211)_sPP_ and (121)_sPP_ reflections of form I of sPP at 2θ = 12.2°, 16°, 18.8° and 20.7° and the (110)_PE_ and (200)_PE_ reflections at 2θ = 21.4° and 23.9° of the orthorhombic form of PE are indicated.

The X-ray diffraction profiles of the sample PE-*b*-sPP recorded at different temperatures during heating and cooling from the melt down to room temperature, are reported in Figure 4. The diffraction profiles of Figure 4A, recorded during first heating of the as-prepared sample, and of Figure 4C, recorded after cooling from the melt during heating of the melt-crystallized sample, show a decrease of the intensity of the diffraction peaks at 2θ = 21° and 24°, corresponding to the 110 and 200 reflections of PE, at temperatures higher than 120–125 °C (profiles e–g of Figure 4A and e–h of Figure 4C), while the intensities of the 200 and 020 reflections of sPP at 2θ = 12 and 16°, respectively, do not change up to 140 °C. This clearly indicates that crystals of PE melt at low temperatures and confirms that the peak at 124 °C in the DSC heating curves of Figure 3a,c corresponds to the melting of PE and the peak at 137 °C corresponds to the melting of sPP.

The diffraction profiles recorded during cooling form the melt at 150 °C to room temperature of Figure 4B indicate that sPP and PE crystallize almost simultaneously, according to the single crystallization peak observed in the DSC cooling curve of Figure 3b, although the 200, 020 and 121 reflections of sPP at 2θ = 12, 16 and 20.7° appear first, already at 120 °C (profile c of Figure 4B), before the 110 and 200 reflections of PE that are well visible only at 115 °C, along with all reflections of sPP (profile d of Figure 4B). Therefore, during the slow cooling and the isothermal necessary to record the diffraction profile, sPP crystallizes first at high temperatures (nearly 120 °C). The intensities of reflections of both sPP and PE increase and become sharper upon further cooling and, as discussed above (Figure 2b), the 211 reflection at 2θ = 18.8° of the ordered form I of sPP develops (profiles e–i of Figure 4B).

The possible phase separation in the melt and the possible formation of phase separated structures for PE-*b*-sPP BCPs has been discussed in the ref [31]. According to mean-field theory, the order–disorder transition for symmetric BCPs occurs at a fixed interaction strength for calculated values of χ*N* = 10.5, where χ is the Flory–Huggins interaction parameter and *N* is the total number of equivalent segments that constitute the macromolecules of the blocks of the BCP [31]. For non-symmetric BCPs the phase separation transition occurs for higher values of χ*N*. For polyolefin-based BCPs, the equivalent segments are assumed as a portion of chains having the density of four CH_2_ units (four carbon atoms segment). The Flory–Huggins interaction parameter χ between sPP and PE has been determined in [31] as: χ = 6.2/*T* − 0.0053, with *T* the absolute temperature. For the sample PE-*b*-sPP with total *M*_n_ = 22,000 and *f*_PE_ = 13%, the total number of equivalent segments *N* that constitute the macromolecules of the blocks is *N* = *M*_n_/56 = 393 (where 56 is the molecular mass of the four CH_2_ carbon atoms segment). Therefore, for this sample the order–disorder transition temperature *T*_ODT_ may be calculated from χ*N ≥* 10.5 = (6.2/*T* − 0.0053)393 and is expected to be lower than 0 °C. This indicates that crystallization of the sample PE-*b*-sPP most likely takes place from a homogeneous melt.

Thin films (thickness lower than 50 nm) of the sample PE-*b*-sPP have been epitaxially crystallized onto the (001) surfaces of crystals of *p*-terphenyl (3Ph) and benzoic acid (BA). Epitaxial crystallization of PE and sPP homopolymers onto crystals of various organic substances has been well-described and used as a tool for growing in thin films crystals of various polymorphic forms with single-crystal or fiber-like orientations [28,33,34,35,36,42]. Polymer–polymer epitaxy, involving heteroepitaxy of sPP with PE and homoepitaxy has been also described [43]. Epitaxial crystallizations of sPP and PE blocks when they are parts of crystalline/amorphous or crystalline–crystalline block copolymers have also been studied [7,15,16,17,18,19,23,24].

**Figure 5:**
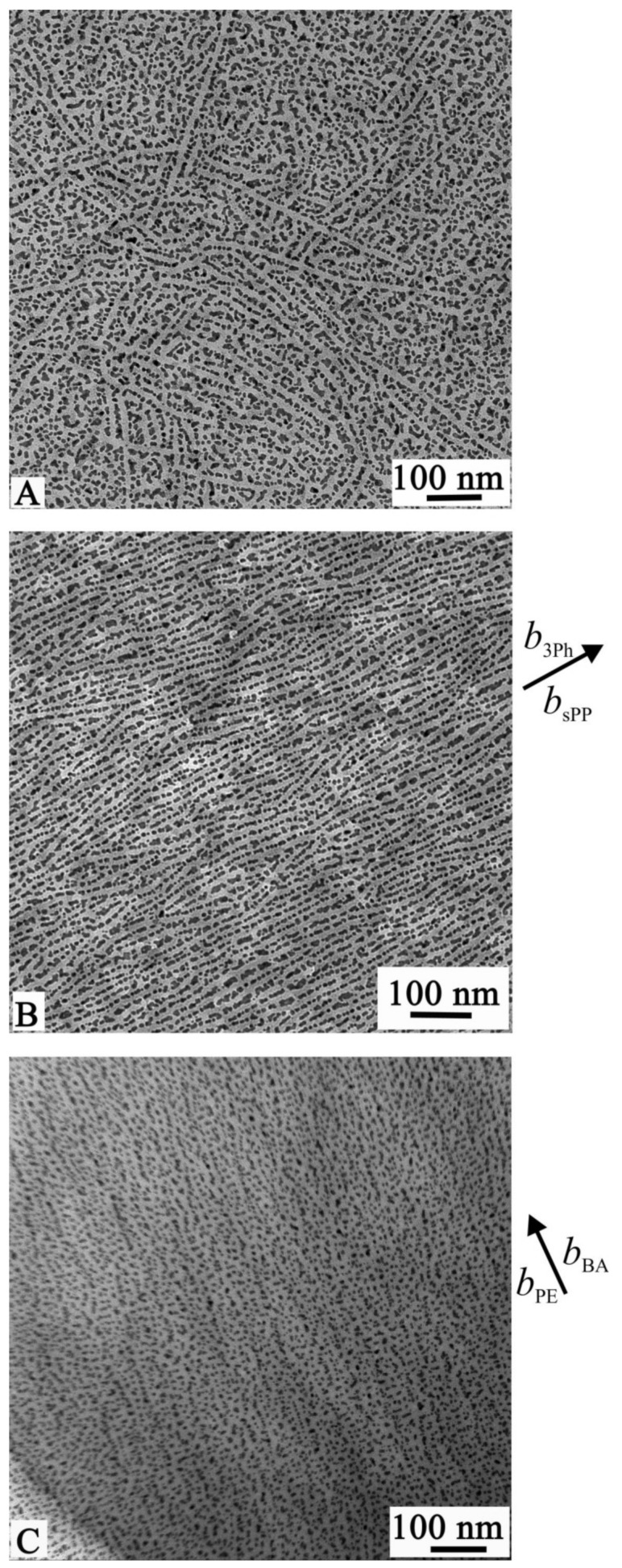
TEM bright-field images of thin films of the sample PE-*b*-sPP with *f*_PE_ = 13% crystallized by simple solution casting without epitaxy (**A**) and epitaxially crystallized on the (001) surface of crystals of 3Ph (**B**) and BA (**C**).

The TEM bright-field images of thin films of the sample PE-*b*-sPP crystallized by simple casting from the polymer solution (without epitaxy) and of films epitaxially crystallized onto 3Ph and BA are reported in Figure 5. The films have been coated with gold particles to improve the contrast in the TEM observation and reveal details of the morphology. The technique of gold decoration is used to visualize edge-on crystalline lamellae of polymers in TEM bright-field images, especially in the case of low TEM amplitude contrast between amorphous and crystalline phases, and to obtain a reliable value of the lamellar periodicity [44,45]. The vaporized gold gathers, indeed, in the ditches made by the interlamellar amorphous material and produces a regular pattern of gold particles, which is observed in bright-field imaging [44,45,46]. In the case of homopolymers this generally produces thin layers of gold particles at the interface between amorphous and crystalline lamellae, containing rows of essentially one gold particle thickness [44,45].

In all the images of Figure 5 the dark spots correspond to the gold particles that presumably are located in the amorphous intra-lamellar phases of PE and sPP, that is, in between the crystalline domains of PE or sPP, whereas the brighter regions correspond to PE and/or sPP crystalline lamellae. It is apparent that in the case of the films crystallized without epitaxy in Figure 5A, the PE and sPP crystalline lamellae (the light stripes) are randomly oriented and are not distinguishable. In the TEM images of the films epitaxially crystallized onto 3Ph (Figure 5B) and BA (Figure 5C), the crystalline lamellae of PE or sPP are in both cases highly aligned along one direction and oriented edge-on on the substrate surface. The epitaxy produces a highly aligned lamellar structure with long crystalline sPP and/or PE lamellae, with average thicknesses of 15 nm.

A single orientation of sPP lamellae on 3Ph and of PE lamellae on BA has been found for the two homopolymers [35,36] and also in epitaxially crystallized crystalline-amorphous BCPs, as in the case of sPP-*b*-EP [18] and PE-*b*-EP [15,19] (EP being an ethylene-propylene amorphous random copolymer). More complex morphology is instead expected for the crystallization of PE onto 3Ph, for which two different orientations of PE lamellae have been observed in the case of PE homopolymer crystallized onto 3Ph [33].

However, thanks to the use of different substrates, the images of Figure 5B,C, although very similar in term of induced single orientation of crystalline lamellae (sPP and PE), reveal the sequence of crystallization events during cooling from the melt and what is the dominant event that drives the final morphology. This information can be, indeed, revealed through interpretation of the images of Figure 5B,C and from the epitaxial relationships between polymer crystals and substrates crystals. The complex morphologies generated in the epitaxial crystallization of the sample PE-*b*-sPP result from interactions between all three components involved, sPP, PE and the crystalline substrate (3Ph or BA).

Both PE and sPP crystallize epitaxially onto crystals of 3Ph [28,33,34,36], and only PE crystallizes epitaxially onto BA [7,15,19,24,28,34,35], whereas no epitaxy exists for sPP onto BA. Epitaxial crystallization produces single crystal-like orientation of PE and sPP crystals onto the (001) exposed face of 3Ph [28,33,34,36] and of PE crystals onto the (001) face of BA crystals [34,35].

**Figure 6:**
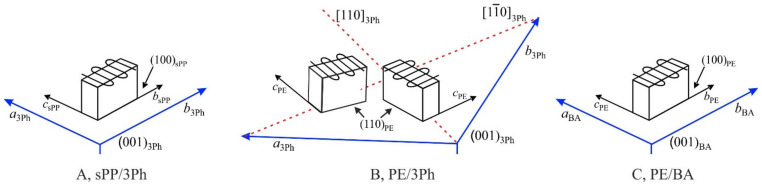
Schemes of the single orientation of crystalline lamellae of sPP (**A**) and double orientations of PE lamellae (**B**) onto the (001) face of 3Ph and of the single orientation PE lamellae onto the (001) face of BA (**C**), induced by epitaxial crystallization [33,35,36].

For sPP onto 3Ph, the (100) plane of crystals of form I of sPP is in contact with the (001) plane of 3Ph; therefore, the crystalline sPP lamellae stand edge-on on the substrate surface, oriented with the *b* and *c* axes of sPP parallel to the *b* and *a* axes of 3Ph, respectively (Figure 6A) [36]. The chain axis of the crystalline sPP lamellae lies flat on the substrate surface and oriented parallel to the *a* axis of 3Ph crystals (Figure 6A). This epitaxy is well explained in terms of the crystal structures of 3Ph (unit cell with *a* = 8.05Å, *b* = 5.55 Å, *c* = 13.59 Å, β = 91.9°) [36] and form I of sPP (orthorhombic unit cell with axes *a* = 14.5 Å, *b* = 5.6 Å or 11.2 Å, *c* = 7.4 Å) [38,39] and matching of the *a*_3Ph_ = 8.05 Å and *b*_3Ph_ = 5.55 Å axes of 3Ph with the *c* = 7.4 Å and *b* = 5.6 Å axes, respectively, of form I of sPP [36]. The epitaxial relationships between sPP and 3Ph crystals are, therefore, (Figure 6A):

(100)_sPP_//(001)_3Ph_

*b*_sPP_//*b*_3Ph_; *c*_sPP_//*a*_3Ph_

In the case of PE/3Ph epitaxy, two different equivalent orientations of PE crystalline lamellae are generated by crystallization onto the (001) face of 3Ph (Figure 6B) [33]. The (110) plane of PE is in contact with the (001) plane of 3Ph [33]. The PE lamellae stand edge-on with the chain axes oriented parallel to the [110] and [1$\bar{1}$0] directions of the 3Ph crystal about 74° apart, as shown in the scheme of Figure 6B. This epitaxy and the selection of the (110) plane as contact plane with the (001) plane of 3Ph is due to the matching between the 4.45 Å interchain distance in the (110) plane of PE and the 4.60 Å interplanar distance of the {110} planes of 3Ph [33]. The epitaxial relationships between sPP and 3Ph crystals are, therefore, (Figure 6B):

(110)_PE_//(001)_3Ph_

*c*_PE_*/*/[110]_3Ph_ and//[1$\bar{1}$0]_3Ph_

Therefore, the epitaxial crystallization of the sample PE-*b*-sPP onto 3Ph should give oriented overgrowth of both crystals of sPP and PE, with a single orientation of sPP lamellae (Figure 6A) and a double orientation of PE lamellae (Figure 6B) onto the (001) surface of the 3Ph substrate [23].

In the case of PE/BA epitaxy, a single orientation of PE lamellae is generated by crystallization of PE onto the (001) face of BA (Figure 6C). The chain axis of the crystalline PE lies flat on the substrate surface and oriented parallel to the *a* axis of BA crystals, as in the case of the PE homopolymer [35]. The (100) plane of PE is in contact with the (001) plane of BA [35]; therefore, the crystalline PE lamellae stand edge-on on the substrate surface, oriented with the *b* and *c* axes of PE parallel to the *b* and *a* axes of BA, respectively [35]. This epitaxy has been well explained in term of matching of the *b* = 4.93 Å and *c* = 2.53 Å axes of the unit cell of PE orthorhombic form (*a* = 7.40 Å, *b* = 4.93 Å, *c* = 2.53 Å) [40] with the *b* = 5.14 Å and *a* = 5.52 Å axes, respectively, of the BA unit cell (*a* = 5.52 Å, *b* = 5.14 Å, *c* = 21.9 Å, β = 97°) [35]. The epitaxial relationships between PE and BA crystals are, therefore, (Figure 6C):

(100)_PE_//(001)_BA_

*b*_PE_//*b*_BA_; *c*_PE_//*a*_BA_

Therefore, based on the epitaxial relationships found for sPP and PE homopolymers onto 3Ph and BA in Figure 6, a single orientation of sPP lamellae on 3Ph and of PE lamellae on BA and a double orientation of PE lamellae onto 3Ph would be expected in the epitaxial crystallization of the PE-*b*-sPP block copolymer. Moreover, no preferential orientation of sPP crystals onto BA is expected. The TEM images of films of the sample PE-*b*-sPP epitaxially crystallized onto 3Ph (Figure 5B) and BA (Figure 5C), instead, clearly show that a single orientation of crystalline lamellae (PE and/or sPP) is obtained onto both 3Ph and PE.

**Figure 7:**
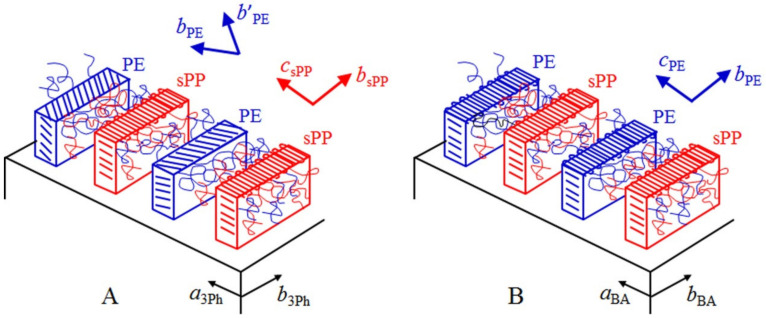
Models of the structures and morphologies that develop upon epitaxial crystallization of PE-*b*-sPP with *f*_PE_ = 13% onto the (001) surfaces of crystals of 3Ph (**A**) and BA (**B**). In A sPP crystallizes first onto 3Ph, forming lamellae aligned with the *c* and *b* axes of sPP parallel to the *a* and *b* axes of 3Ph. PE crystallizes after sPP in the confined inter-lamellar regions prescribed by the oriented sPP lamellae (**A**). In B PE crystallizes first onto BA forming lamellae aligned with the *c* and *b* axes of PE parallel to the *a* and *b* axes of BA. sPP crystallizes after PE in the confined inter-lamellar regions prescribed by the oriented PE lamellae (**B**).

In the case of the crystallization onto 3Ph of Figure 5B, the obtained single orientation of the crystalline lamellae and the absence of double oriented lamellae of PE, as in Figure 6B, indicate that the observed parallel lamellae oriented along one direction are of the sPP blocks that, based on the Figure 6A, must have a single orientation with the *c* axis of sPP parallel to the *a* axis of 3Ph. The crystallization of the sPP block with the expected single lamellae orientation, therefore, defines the overall morphology of the whole epitaxially crystallized film with evident crystalline lamellae oriented along only one direction (Figure 5B). This means that sPP must have crystallized first. None of the expected PE lamellae with two different orientations 74° apart (Figure 6B) are visible. Therefore, PE crystallizes after sPP in the confined inter-lamellar regions prescribed by the oriented sPP lamellae. These trapped and thin PE lamellae are hardly visualized by the gold decoration. The final morphology (Figure 5B) is, therefore, driven by the crystallization of sPP, in agreement with the fact that the sPP block is longer than the PE block and according with the X-ray diffraction data of Figure 4B that have indicated that sPP crystallizes first upon cooling from the melt. A scheme of the final morphology representing the TEM image of Figure 5B is shown in Figure 7A. PE lamellae are confined between sPP lamellae and follow the orientation of the sPP lamellae that are aligned with the *c* and *b* axes of sPP parallel to the *a* and *b* axes of 3Ph, respectively. Since the growth of PE is confined between sPP lamellae and the epitaxy should produce different orientations of PE chain axes parallel to the [110] and [1$\bar{1}$0] directions of 3Ph (Figure 6B), it is most probable that PE lamellae are parallel to the sPP lamellae but are made of chains tilted with respect to their basal fold surface, as shown in the model of Figure 7A. The tilting of PE chains with tilt angle of 45° to the lamellar normal has already been described [47,48,49]. Thus, in these systems, the stem orientation is dictated by the epitaxy with 3Ph, but the fold surface orientation is dictated by the orientation of the lamellae of the block that crystallizes first (sPP). Therefore, in the confined sPP interlamellar regions, the trapped PE lamellae are parallel to the sPP lamellae and oriented along the direction dictated by the sPP crystallization, with the PE chains tilted at 74/2 = 37° to the lamellar normal (Figure 7A).

The epitaxial crystallization of PE-*b*-sPP onto BA also produces single orientation of crystalline lamellae aligned along one direction, the *b* axis of BA (Figure 5C). Since no epitaxy exists for crystallization of sPP onto BA, random orientation of sPP lamellae is expected, as in Figure 5A for crystallization of PE-*b*-sPP without substrate. This random orientation is not observed in the morphology of Figure 5C. Therefore, the obtained single orientation of the crystalline lamellae and the absence of random orientation of sPP lamellae indicate that the observed parallel lamellae oriented along one direction are of the PE blocks that, based on Figure 6C, must have a single orientation, with the *c* and *b* axes of PE parallel to the *a* and *b* axes of BA, respectively. The crystallization of the PE block with the expected single lamellae orientation, therefore, defines the overall morphology of the whole epitaxially crystallized film with evident crystalline lamellae oriented along only one direction (Figure 5C). This may be explained considering that, even though the sPP block crystallizes first in the absence of substrates (Figure 4B) or onto 3Ph, the PE block must have crystallized first in the presence of BA, or nearly contemporarily to the sPP block. Therefore, sPP crystallizes after PE (or with PE) in the confined interlamellar regions prescribed by the oriented PE lamellae. However, since the epitaxial crystallization of the polymer blocks onto BA is preceded by the directional solidification of BA [7] that induces alignment of the BCP microdomains along the *b* axis of BA (the growth front direction) before and during the solidification and crystallization of the BCP, the process results in alignment of both PE and sPP crystalline lamellae parallel to the *b* axis of BA. Then, epitaxy of PE onto BA produces alignment of the *c* and *b* axes of PE parallel to the *a* and *b* axes of BA, respectively. A scheme of the final morphology representing the TEM image of Figure 5C is shown in Figure 7B. sPP lamellae are confined between PE lamellae and follow the orientation of the PE lamellae. Since there is no preferred orientation of the *c* axis of sPP onto BA, it is probable that sPP lamellae are parallel to the PE lamellae with the chains normal to their basal fold surface and parallel to the chain axis of PE, that is, the stem orientation dictated by the epitaxy of PE onto BA (Figure 7B).

## 4. Conclusions

A sample of crystalline–crystalline PE-*b*-sPP block copolymers with 13% volume fraction of the PE block was synthesized with a stereoselective living organometallic catalyst. The structure and morphology of the PE-*b*-sPP block copolymer have been studied in the bulk and in thin films epitaxially crystallized on crystals of 3Ph and BA substrates.

In both as-prepared and melt-crystallized samples of PE-*b*-sPP the sPP block crystallizes in the stable form I and the PE block crystallizes in the orthorhombic form. Crystals of PE and sPP melt at different temperatures, at 124 °C and 137 °C, respectively. The two blocks crystallize from the melt by cooling at a controlled rate (10 °C/min) almost simultaneously, and only one exothermic peak is observed in the DSC cooling curve. However, diffraction profiles recorded during cooling have demonstrated that the longer sPP block crystallizes first.

Thin films of the sample PE-*b*-sPP were epitaxially crystallized onto the (001) surfaces of crystals of *p*-terphenyl (3Ph) and benzoic acid (BA). The complex morphologies generated in the epitaxial crystallization result from interactions between all three components involved, sPP, PE and the crystalline substrate (3Ph or BA). The epitaxial crystallization produces oriented growth of both crystals of sPP and PE depending on the substrate, with a single orientation of sPP lamellae onto the (001) surface of 3Ph crystals and a single orientation of PE lamellae onto the (001) surface of BA, according to the epitaxy of sPP with 3Ph and PE with BA. Epitaxy of PE with 3Ph should instead produce a double orientation of PE lamellae onto the (001) surface of 3Ph. The process also produces development of ordered nanostructures composed of alternating lamellar domains of PE and sPP, guided by the orientation of the sPP or PE crystalline lamellae.

TEM bright-field images provide details of the resulting morphology and reveal the sequence of the crystallization events. In the case of the crystallization of PE-*b*-sPP onto 3Ph, highly oriented crystalline lamellae aligned along one direction are obtained. The expected double orientation of PE lamellae onto the (001) surface of 3Ph is not observed. This indicates that sPP crystallizes first and defines the overall morphology of the whole epitaxially crystallized film, forming, according to epitaxy onto 3Ph, long lamellae oriented along one direction with the *c* and *b* axis of sPP parallel to the *a* and *b* axes of 3Ph, respectively. PE crystallizes after sPP in the confined inter-lamellar regions prescribed by the oriented sPP lamellae.

The epitaxial crystallization of PE-*b*-sPP onto BA also produces single orientation of crystalline lamellae aligned along one direction, the *b* axis of BA. Since no epitaxy exists for crystallization of sPP onto BA, random orientation of sPP lamellae would be expected. Therefore, the obtained single orientation of the crystalline lamellae and the absence of random orientation of sPP lamellae indicate that the observed parallel lamellae oriented along one direction are of the PE blocks that, according to the epitaxy of PE with BA, must have a single orientation with the *c* and *b* axes of PE parallel to the *a* and *b* axes of BA, respectively. The crystallization of the PE block defines the overall morphology of the whole epitaxially crystallized film. This may be explained considering that the PE block must have crystallized first in the presence of BA, or nearly contemporarily to the sPP block. Therefore, sPP crystallizes after PE (or with PE) in the confined inter-lamellar regions prescribed by the oriented PE lamellae. However, since the epitaxial crystallization of the polymer blocks onto BA is preceded by the directional solidification of BA that induces alignment of the BCP microdomains along the *b* axis of BA (the growth front direction) before and during the solidification and crystallization of the BCP, the process results in alignment of both PE and sPP crystalline lamellae parallel to the *b* axis of BA. Then, epitaxy of PE onto BA produces alignment of the *c* and *b* axes of PE parallel to the *a* and *b* axes of BA, respectively.

These data show that the use of epitaxial crystallization and the choice of suitable substrate offer a means to produce oriented nanostructures and morphologies of BCP depending on the BCP composition and the substrates.

## Data Availability

The data in this study are available on reasonable request from the corresponding author.
